# Efficacy and safety of combined immunotherapy and antiangiogenesis with or without chemotherapy for advanced non-small-cell lung cancer: A systematic review and pooled analysis from 23 prospective studies

**DOI:** 10.3389/fphar.2022.920165

**Published:** 2022-08-10

**Authors:** Ruo-Lin Gao, Jun Song, Li Sun, Zhi-Xuan Wu, Xiao-Fang Yi, Shu-Ling Zhang, Le-Tian Huang, Jie-Tao Ma, Cheng-Bo Han

**Affiliations:** Department of Oncology, Shengjing Hospital of China Medical University, Shenyang, China

**Keywords:** non-small cell lung cancer, immunotherapy, angiogenesis inhibitors, combination therapy, chemotherapy

## Abstract

**Purpose:** Immune checkpoint and antiangiogenic inhibitors have a potentially synergistic antitumor effect. We aimed to assess the efficacy and safety of immunotherapy in combination with antiangiogenesis therapy with or without chemotherapy in patients with advanced non-small-cell lung cancer (NSCLC).

**Methods:** PubMed, Embase, the Cochrane library, Google Scholar, Ovid, Scopus, and Web of Science were searched for eligible trials. ClinicalTrials.gov and meeting abstracts were also searched for qualified clinical studies. The inclusion criteria were as follows: prospective studies (including single-arm studies) that evaluated efficacy and/or toxicity of immunotherapy combined with antiangiogenic agents (A + I) with or without chemotherapy (A + I + chemo) in patients with advanced or metastatic NSCLC; and primary outcome of each study reported at least one of these endpoints: progression-free survival (PFS), overall survival, objective response rate (ORR), disease control rate (DCR), or adverse events (AEs).

**Results:** Twenty three prospective studies comprising 1,856 patients with advanced NSCLC were included. The pooled ORR, median PFS and estimated overall survival were 39%, 6.8 months [95% confidence interval (CI), 5.53–8.13], and 18.6 months in the overall group. Similar ORR and median PFS with A + I + chemo versus A + I were observed in patients treated in first-line setting [59% and 9.47 months (95% CI, 6.45–12.49) versus 52% and 10.9 months (95% CI, 1.81–19.98), respectively]. We also observed improved ORR and mPFS with A + I + chemo versus A + I in subsequent-line setting [56% and 8.1 months (95% CI, 5.00–11.26) versus 22% and 5.1 months (95% CI, 4.01–6.15), respectively]. Efficacy of A + I + chemo therapy was evident across different PD-L1 subgroups, especially in patients with EGFR mutations [ORR: 59%; mPFS: 8.13 months (95% CI: 5.00–11.26)] or baseline liver metastases. The incidence of AEs with a major grade of ≥3 in the overall, A + I, and A + I + chemo groups were 4.1% vs. 5.5% vs. 3.4% for proteinuria, 13.7% vs. 16.2% vs. 9.7% for hypertension, and 1.9% vs. 1.2% vs. 2.8% for rash, respectively. No new safety signals were identified in this pooled analysis.

**Conclusion:** Immunotherapy combined with antiangiogenic agents with or without chemotherapy showed encouraging antitumor activity and an acceptable toxicity profile in treatment-naïve or pretreated patients with advanced NSCLC. Doublet treatment with immunotherapy and antiangiogenic agents might be a new option for patients with advanced NSCLC, especially those who are treatment-naive or cannot tolerate chemotherapy.

## Introduction

Lung cancer is the leading cause of cancer-related deaths worldwide. Non-small-cell lung cancer (NSCLC) accounts for approximately 85% of all lung cancer cases. It is often diagnosed at a late stage and has a poor prognosis (Siegel et al., 2022). The emergence of immunotherapy has dramatically changed the treatment landscape for patients with NSCLC. Programmed cell death protein-1 (PD-1) or its ligand 1 (PD-L1) immune checkpoint inhibitors (ICIs) have been in the forefront of this breakthrough. Data from the KEYNOTE-024 study showed a five-year overall survival (OS) of 32% for patients with PD-L1 tumor proportion score (TPS) of ≥50% who were treated with pembrolizumab, which was twice the value observed in the platinum-based chemotherapy alone group (16%) (Reck et al., 2019a). Currently, a variety of PD-1/PD-L1 ICIs are approved for the treatment of advanced NSCLC. A hallmark of drugs with the PD-1/PD-L1 axis is the induction of deep and durable antitumor responses that can translate into a survival benefit in patients with a variety of tumor histologies (Tumeh et al., 2014; Garon et al., 2015; Overman et al., 2018). However, long-term responses are restricted to a minority of patients from single-agent anti-PD-1/PD-L1 therapy (Rittmeyer et al., 2017; Garon et al., 2019; Mok et al., 2019), highlighting an unmet need to develop novel combination strategies.

In recent years, researchers have been focusing on the use of immunotherapy as a basic therapy in combination with other treatment strategies, including radiotherapy, chemotherapy, and targeted drugs, which are thought to enhance tumor-associated immunogenicity by inducing tumor cell death and the release of new antigens (Pilotto et al., 2015; Tan et al., 2016). Antiangiogenesis therapy is another promising strategy that mainly blocks the vascular endothelial growth factor (VEGF)/VEGF receptor (VEGFR) signaling pathway, which is involved in the process of tumorigenesis, development, and metastasis, as well as the regulation of tumor microenvironment (Dvorak, 2015). Tumor neo-angiogenesis and immune-escape are interconnected processes (Pivarcsi et al., 2007). The irregular tumor blood vessels enable immune evasion and decrease anti-cancer therapy efficacy by limiting the transportation of oxygen and cytotoxic T cells from the bloodstream to the tumor environment (Siemann, 2011). As consequence, the resulting hypoxia induces the upregulation of immune checkpoints, as well as the infiltration of immunosuppressive components, such as regulatory T cells and myeloid-derived suppressor cells within the tumor microenvironment (Fares et al., 2019). Antiangiogenic therapies have been found to increase cytotoxic T cell trafficking into tumors, reduce immunosuppressive components, and inhibit Treg proliferation (Terme et al., 2013). In addition, activated immunity by immune checkpoint blockade also facilitates antiangiogenesis by downregulating the expression of VEGF and alleviating hypoxic conditions (Guo and Cui, 2020). Therefore, ICIs and antiangiogenesis therapy could hypothetically have synergistic or additive effects.

Different studies have investigated the combinations of ICIs and antiangiogenic inhibitors, including both monoclonal antibodies (mAbs) targeting VEGF/VEGFR, such as bevacizumab and ramucirumab, and small molecule tyrosine kinase inhibitors (TKIs) (Gadgeel et al., 2018; Reck et al., 2019b; Herbst et al., 2019; Zhou et al., 2019; Bang et al., 2020; Herbst et al., 2020; Lee et al., 2020; Nishio et al., 2020; Seto et al., 2020; Taylor et al., 2020; Zhou et al., 2020; Han et al., 2021a; Ardeshir-Larijani et al., 2021; Han et al., 2021b; Chu et al., 2021; Gao et al., 2021; Leal et al., 2021; Neal et al., 2021; Yang et al., 2021; Gao et al., 2022; Lee et al., 2022; Lu et al., 2022; Ren et al., 2022). However, the reported studies to date are mostly single-arm or retrospective studies with limited patient enrollment and heterogeneous results. Here, we conducted a pooled analysis to evaluate the clinical efficacy and safety of immunotherapy in combination with antiangiogenesis therapy with or without chemotherapy in patients with advanced NSCLC, aiming to generate a more comprehensive understanding and subsequently guide the application of this new combination therapy in clinical practice.

## Methods

### Search strategy

The present systematic review and meta-analysis were conducted in accordance with the Preferred Reporting Items for Systematic Reviews and Meta-Analysis (PRISMA) guidelines. The PICOS (Population, Intervention, Comparison, Outcomes and Study design) system was used to describe the key items for framing the objective and methodology of this review. A comprehensive search of online databases, including PubMed, Embase, the Cochrane library, Google Scholar, Ovid, Scopus, and Web of Science, was performed. ClinicalTrials.gov was also searched for qualified clinical studies. Key search terms included “non-small cell lung cancer,” “immunotherapy,” and “anti-angiogenic inhibitor”. Manual updates for abstracts presented before the 2022 meetings, such as the American Society of Clinical Oncology, European Society for Medical Oncology, World Conference of Lung Cancer, and American Association for Cancer Research, were also performed. Reference lists for the enrolled studies were manually scanned to ensure that all relevant literature was retrieved. The final literature search was performed on 31 May 2022.

### Literature selection criteria

All eligible studies were included in the pooled analysis if they met the following inclusion criteria: 1) prospective studies (including single-arm studies) that evaluated efficacy and/or toxicity of immunotherapy combined with antiangiogenesis therapy with or without chemotherapy in patients with advanced or metastatic NSCLC; 2) the primary outcome of each study reported at least one of these endpoints: progression-free survival (PFS), OS, objective response rate (ORR), disease control rate (DCR), or adverse events (AEs) based on the Common Terminology Criteria for Adverse Events version 3.0 or 4.0; 3) the study report was written in English; and 4) the number of cases in the study was ≥10.

Data obtained from retrospective studies and non-original studies including meta-analysis, commentaries, editorials, and reviews were excluded from our study. Also, unpublished data and presentations that did not provide accurate and clear data on research variables were excluded.

### Data extraction and synthesis

After completing the literature search according to the inclusion criteria, two team members checked the authorship, institutions, and abstracts to exclude duplicate papers. Then, two team members independently extracted data from all eligible studies, including first author information and the publication year; baseline study information, including patient characteristics and therapy methods; median PFS (mPFS) and median OS (mOS); ORR and DCR; and AEs.

### Sensitivity analysis and publication bias

Sensitivity analyses were performed for the ORR results based on the leave-one-out approach. The potential for publication bias in the reported ORR values was assessed using funnel plots and Egger’s test, with the appropriate accuracy intervals. In addition, we undertook the nonparametric trim and fill method, which conservatively imputes hypothetical negative unpublished studies to mirror the positive studies that cause funnel plot asymmetry.

### Statistical methods

Statistical analyses were performed using the Stata 16.0 software (StataCorp LLC, College Station, TX, United States). The data for the main outcomes of each study were pooled and included the ORR, DCR, mPFS, mOS, and AE incidence rate. Subgroup analyses were performed on studies that reported the treatment line and treatment methods. Statistical heterogeneity among the studies was detected using the I^2^ statistic. A random-effects model (DerSimonian-Laird method) was used if the probability (*p*) value was ≤0.05 or I^2^ was >50%, indicating significant heterogeneity. Otherwise, a fixed-effects model (inverse-variance method) was used. A meta-regression was performed to evaluate the effect of age, sample size, sex, Eastern Cooperative Oncology Group (ECOG) performance score, smoking history, and tumor histology being adjusted on the pooled adjusted ORR.

## Results

### Study population

**FIGURE 1:**
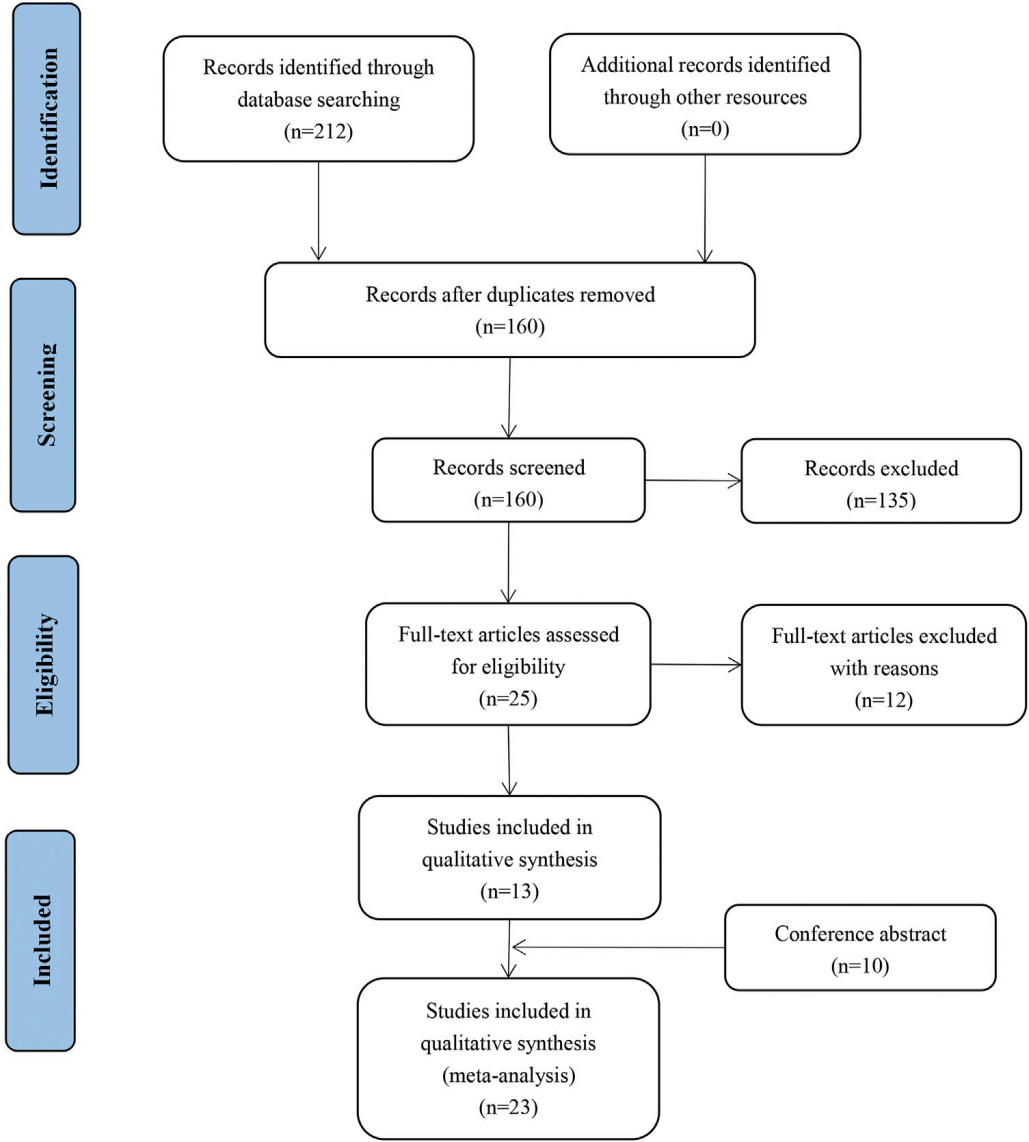
Literature search and selection process flow diagram.

The full texts of 30 published studies and meeting abstracts were reviewed. A PRISMA flow diagram of the literature search process is shown in Figure 1. A total of 23 studies involving 1,856 patients with advanced NSCLC met the inclusion criteria. The included studies comprised three prospective cohort studies, five single-arm prospective studies, and four randomized controlled trials (RCTs) (Table 1). Baseline characteristics of patients from included studies were described in Supplementary Table S7. The pooled analysis assigned patients into two groups according to the therapeutic regimen: antiangiogenic agents combined with ICIs with chemotherapy (A + I + chemo) treatment in six studies with 888 patients; and antiangiogenic agents combined with ICIs without chemotherapy (A + I) treatment in seventeen studies with 968 patients. Patients in the A + I + chemo and A + I groups were further subgrouped according to the treatment line, type of antiangiogenic agents (mAbs or TKIs), ICI type (PD-1 or PD-L1), and EGFR mutation status.

**TABLE 1:** Study characteristics.

Study	Year	Enrolled patient	Design	Tx arm (no. of patients)	Tx line	ORR	DCR	PFS, mos	OS, mos
Reck	2019	Stage IV NS-NSCLC	Phase 3 Randomized	Atezo + PacCb (*n* = 402/) Atezo + Bev + PacCb (*n* = 400) Bev + PacCb (*n* = 400)	1/2	40.6% 56.4% 40.2%	NR	6.7 (NR) 8.4 (8.0–9.9) 6.8 (6.0–7.0)	19.5 (16.3–21.3) 19.8 (17.4–24.2) 14.9 (13.4–17.1)
Zhou	2020	Stage IIIb/IV NS-NSCLC	Phase 1b/2 Single group	Cam + Apa (*n* = 105)	≥2	30.9%	73.3%	5.7 (4.5–8.8)	15.5 (10.9–24.5)
Herbst	2019	Stage IV NSCLC	Phase 1a/b Non-randomized	Pembro + Ram (*n* = 27)	≥2	30%	85%	9.7 (4.6–27.6)	26.2 (11.8–nr)
Herbst	2020	Stage III/IV NSCLC	Phase 1 Non-randomized	Pembro + Ram (*n* = 26)	1	42.3%	84.6%	9.3 (4.0–nr)	nr
Chu	2021	Stage III/IV NSCLC	Phase 1 Non-randomized	Sinti + Anlo (*n* = 22)	1	77.3%	100%	15 (8.3–nr)	nr
Seto	2020	Stage III/IV NSCLC with high PD-L1 expression	Phase 2 Single group	Atezo + Bev (*n* = 39)	1	64.1%	NR	15.9 (5.65–15.93)	nr
Lee	2020	Stage IIIb/IV NS-NSCLC	Phase 3 Randomized	Niv + Bev + PacCb (*n* = 275) Placebo + Bev + PacCb (*n* = 275)	1	61.5% 50.5%	NR	12.1 (9.8–14) 8.1 (7.0–8.5)	25.4 (21.8–nr) 24.7 (20.2–nr)
Taylor	2020	Stage III/IV NSCLC	Phase 1b/2 Single group	Pembro + Lenva (*n* = 21)	≥1	33.3%	80.9%	5.9 (2.3–13.8)	NR
Nishio	2020	Stage IV NS-NSCLC	Phase 3 part 1 Randomized Double-blind	Pembro + Lenva + PemCb/Cis (*n* = 13)	1	69.2%	92.3%	NR	NR
Bang	2020	Stage IIIb/IV NSCLC	Phase 1a/b Non-randomized	Durva + Ram (*n* = 28)	≥2	11%	57%	2.7 (1.6–5.8)	11 (6.2–15.2)
Ardeshir-Larijani	2021	Stage IIII NS-NSCLC	Phase 2 Single group	Atezo + Bev + PemCb (*n* = 30)	1	35.71%	92.85%	NR	NR
Yang	2021	Stage IV NSCLC	Phase 3 Randomized	Pembro + Lenva (*n* = 309) Pembro + Placebo (*n* = 314)	1	40.5% 27.7%	NR	6.6 (6.1–8.2) 4.2 (4.1–6.2)	14.1 (11.4–19.0) 16.4 (12.6–20.6)
Ren	2022	Stage IIIb-IV NS-NSCLC	Phase 1b/2 Single group (cohort 4)	Cam + Apa (*n* = 25)	1	40%	92%	9.6 (5.5–nr)	nr
Han	2021	Stage IIIb-IV NS-NSCLC	Phase 3 Randomized	Penpulimab + Anlo (*n* = 26)	1	57.1%	90.5%	nr	nr
Zhou	2019	Stage IIIb/IV NS-NSCLC	Phase 1/2 Single group (cohort 1)	Cam + Apa (*n* = 96)	≥2	30.8%	82.4%	5.9 (5.5–10.3)	nr
Neal	2021	Stage IV NSCLC	Phase 1b Single group	Atezo + cabozantinib (*n* = 30)	≥2	23%	83%	NR	nr
Leal	2021	Stage III-IV NS-NSCLC	Phase 2 Single group	Nivo + sitravatinib (*n* = 68)	≥2	16%	NR	6	15 (9.3–21.1)
Han	2021	Stage IIIb-IV NSCLC	Phase 3 Randomized	TQ-B2450 (PD-L1)+Anlo (*n* = 68) TQ-B2450 (PD-L1) (*n* = 33)	≥2	30.9% 3%	73.5% 54.6%	6.9 (5.3–12.4) 2.7 (1.4–4.7)	nr nr
Lee	2022	Stage IIIb/IV NSCLC	Phase 2 Single group (stages II)	Atezo + Bev (*n* = 24)	≥3	12.5%	87.5%	5.6 (4.1–7.1)	14 (10.7–17.4)
Gao	2021	EGFR-mutated NSCLC	Phase 1b/2 Single group (cohort 2)	Cam + Apa (*n* = 40)	≥3	20%	62.5%	3.2 (1.5–6.4)	nr
Gao	2022	Stage IIIb/IV non-central squamous NSCLC	Phase 1b/2 Single group (cohort 3)	Cam + Apa (*n* = 25)	≥2	32%	84%	6.0 (3.6–8.3)	12.8 (6.4–nr)
Gadgeel	2018	Stage IIIb/IV NS-NSCLC	Phase 1/2 Single group (cohort B)	Pembro + Bev + PacCb (*n* = 25)	1	56%	76%	7.1 (4.2–14.3)	16.7 (8.5–nr)
Lu	2021	Stage IIIb-IV EGFR-mutated advanced NS-NSCLC	Phase 3 Randomized	Sinti + Bev + PemCs (*n* = 148) Sinti + PemCs (*n* = 145) PemCs (*n* = 151)	≥2	43.9% 33.1% 25.2%	NR NR NR	6.9 (6.0–9.3) 5.6 (4.7–6.9) 4.3 (4.1–5.4)	nr nr nr

Tx, treatment; NS, non-squamous; NSCLC, non-small cell lung cancer; Atezo, atezolizumab; Bev, bevacizumab; PacCb, paclitaxel plus carboplatin; Cam, camrelizumab; Apa, apatinib; Pembro, pembrolizumab; Ram, ramucirumab; Sin, sintilimab; Anlo, anlotinib; Niv, nivolumab; Len, lenvatinib; PemCb, pemetrexed plus carboplatin; Durva, durvalumab; Cis, cisplatin; mos, months; ORR, objective response rate; DCR, disease control rate; PFS, progression-free survival; OS, overall survival; NR, not reported; nr, not reached.

### ORR, DCR, and DOR

**FIGURE 2:**
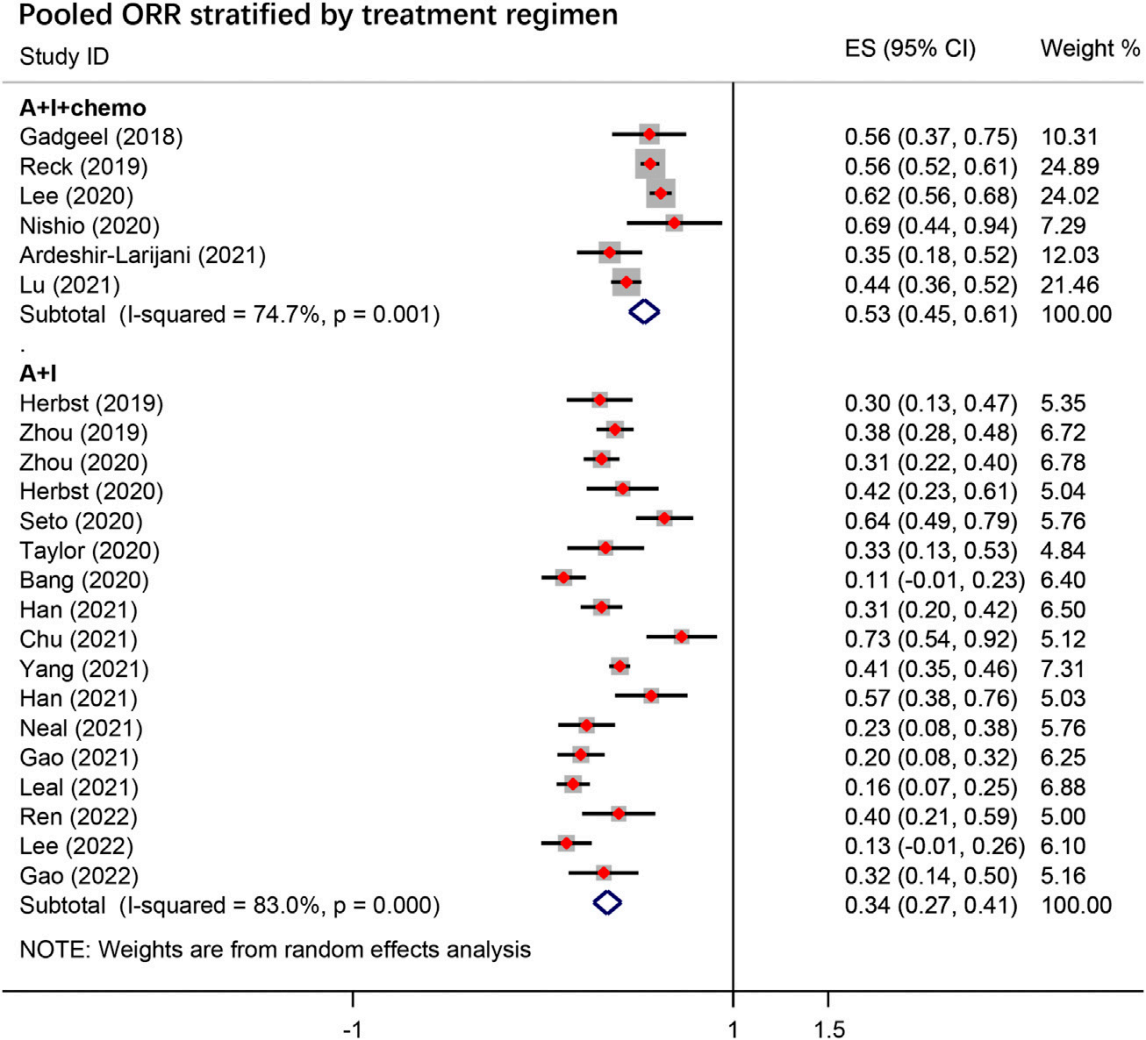
The pooled objective response rate (ORR) in the overall group stratified by treatment regimen. A + I + chemo group: antiangiogenic agents combined with ICIs with chemotherapy; A + I group: antiangiogenic agents combined with ICIs.

The pooled overall ORR for A + I ± chemo from 23 studies was 39.0% [95% confidence interval (CI), 36.0–55.0], with 53.0% (95% CI, 47.8–64.7) in the A + I + chemo group and 34.0% (95% CI, 28.0–52.0) in the A + I group (Figure 2). The pooled DCR was 83.0% overall, 89.0% in the A + I + chemo group, and 81.0% in the A + I group. The subgroup analysis revealed a significant difference in the ORR of patients receiving A + I treatment in first-line settings vs. subsequent-line setting, and the values were 52.0% and 22.0%, respectively. No significant difference in ORR was observed in other A + I subgroups stratified according to type of antiangiogenic agents (mAbs 31% vs. TKIs 35%), ICI type (Anti-PD-1 37% vs. Anti-PD-L1 28%), and EGFR mutation status (EGFR+ 32% vs. EGFR− 34%). The detailed results are summarized in Table 2; Supplementary Table S1.

**TABLE 2:** Objective response rate (ORR) for combined immunotherapy and antiangiogenesis therapy with or without chemotherapy.

Group	No. of studies	No. of patients	Pooled values (95% CI), %
Overall	23	1,856	39.0 (31.0–47.0)
A + I	17	968	34.0 (26.0–42.0)
A + I + chemo	6	888	53.0 (45.0–61.0)
First-line therapy			
A + I	6	447	52.0 (40.0–64.0)
A + I + chemo	5	696	59.0 (51.0–66.0)
Subsequent-line therapy			
A + I	10	500	22.0 (17.0–28.0)
A + I + chemo	2	182	56.0 (30.0–83.0)
Anti-PD-1 therapy			
A + I	12	779	37.0 (28.0–45.0)
A + I + chemo	4	461	56.0 (44.0–68.0)
Anti-PD-L1 therapy			
A + I	5	189	28.0 (11.0–45.0)
A + I + chemo	2	427	54.0 (19.0–89.0)
Antiangiogenic TKIs			
A + I	6	824	35.0 (26.0–43.0)
A + I + chemo	1	13	69.0 (44.0–94.0)
Antiangiogenic mAbs			
A + I	11	144	31.0 (11.0–52.0)
A + I + chemo	5	727	55.0 (47.0–67.0)
EGFR mutation-positive			
A + I	1	25	32.0 (14.0–50.0)
A + I + chemo	2	182	56.0 (30.0–83.0)
EGFR mutation-negative			
A + I	16	943	34.0 (26.0–41.0)
A + I + chemo	5	696	59.0 (51.0–66.0)

95% CI, 95% confidence interval; A + I + chemo, antiangiogenic agents combined with ICIs with chemotherapy; A + I, antiangiogenic agents combined with ICIs; ICIs, immune checkpoint inhibitors; Anti-PD-1, programmed cell death protein-1 inhibitor; Anti-PD-L1, programmed cell death ligand-1 inhibitor; mAbs, monoclonal antibodies; TKIs, small molecule tyrosine kinase inhibitors; ORR, objective response rate.

Of the 23 studies analyzed, three subsequent-line and one first-line studies in the A + I group involving 183 patient reported subgroup efficacy analysis of ORR according to the PD-L1 expression level in tumors, which resulted in a pooled ORR of 47% for the PD-L1-positive tumor and 28% for the PD-L1-negative tumor.

Eight studies recorded the DOR data, with the 95% CI upper limit unreached in three of them, so that the pooled median DOR was calculated using a weighted average of the single-study medians. Median DOR estimates computed using Ûj (Û1, Û2, Û3, Û4, Û5) were obtained in five eligible studies, with group sizes calculated utilizing N_j_ (N_1_, N_2_, N_3_, N_4_, N_5_). These were summed to yield N_all_. The pooled median DOR was then estimated as the group-size weighted average as follows: Ûall = (1/Nall) ∑ Nj × Ûj (Sun et al., 2020). The last estimated pooled DOR was 11.4 months.

### Survival

#### PFS and OS

**FIGURE 3:**
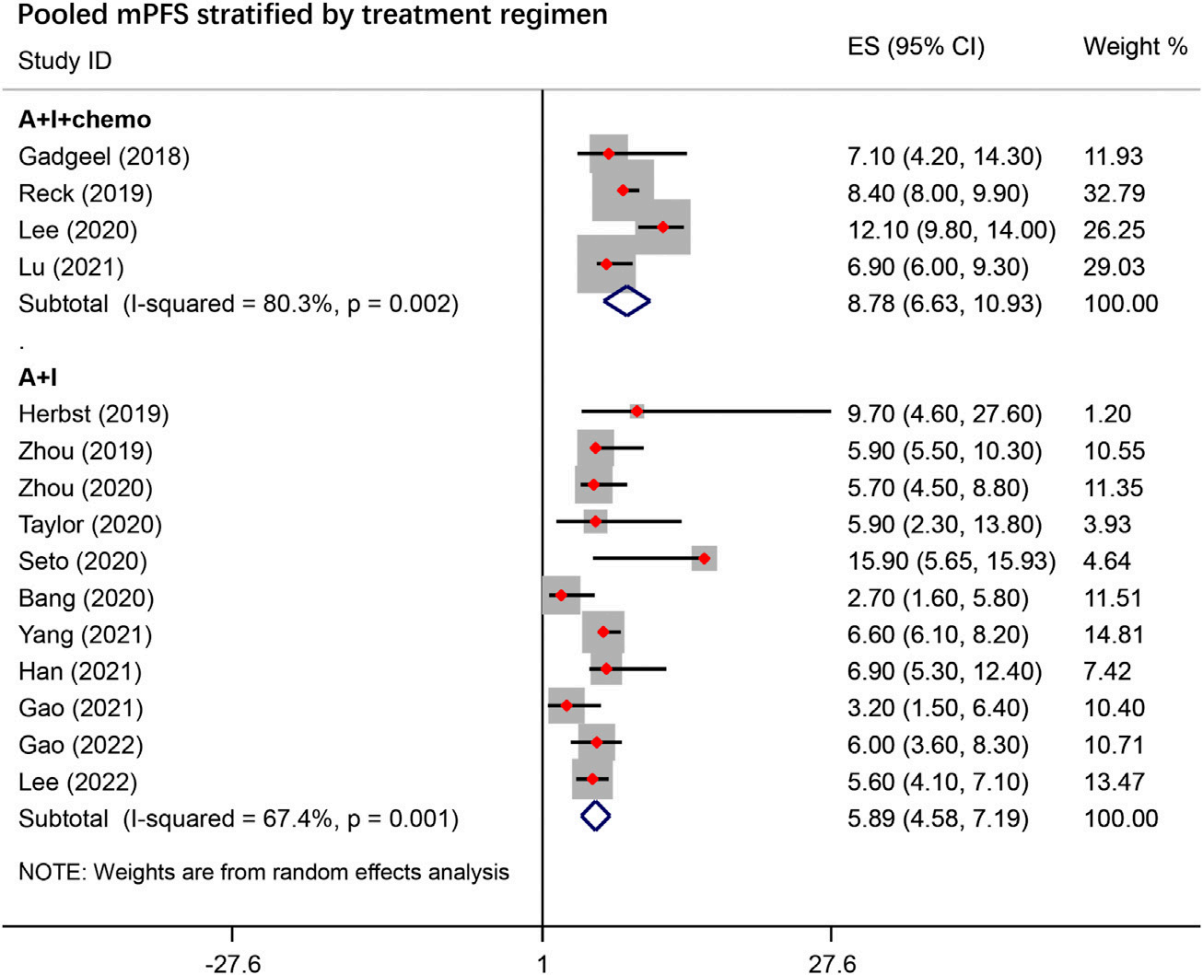
Pooled median progression-free survival (mPFS) stratified by treatment regimen. A + I + chemo group: antiangiogenic agents combined with ICIs with chemotherapy; A + I group: antiangiogenic agents combined with ICIs.

The pooled survival data are summarized in Table 3. The pooled mPFS was 6.83 months (95% CI, 5.53–8.13) overall, 8.78 months (95% CI, 6.63–10.93) with A + I + chemo treatment and 5.89 months (95% CI, 4.58–7.19) with A + I treatment (Figure 3). In the A + I treatment, the mPFS was 10.9 months (95% CI, 1.81–19.98) in the first-line subgroup and 5.08 months (95% CI, 4.01–6.15) in the subsequent-line subgroup. Subgroup analysis of A + I treatment showed a mPFS of 5.90 (95% CI, 5.00–6.79) months compared to 7.07 months (95% CI, 3.41–10.72) in the Anti-PD-1 and Anti-PD-L1 inhibitor subgroups, respectively, and 5.95 months (95% CI, 5.11–6.80) compared to 7.51 months (95% CI, 3.41–11.88), in the TKI and mAbs subgroups, respectively. In the A + I + chemo group, no significant difference in mPFS was observed in subgroups stratified according to ICI types (Anti-PD-1 14.57 months vs. Anti-PD-1 12.89 months), and treatment line (first-line 9.47 months vs. subsequent-line 8.13 months).

**TABLE 3:** Median progression-free survival (mPFS) for combined immunotherapy and antiangiogenesis therapy with or without chemotherapy.

Group	No. of studies	No. of patients	Pooled PFS (95% CI), months
Overall	15	1,438	6.83 (5.53–8.13)
A + I	11	782	5.89 (4.58–7,19)
A + I + chemo	4	656	8.78 (6.63–10.93)
First-line therapy			
A + I	2	584	10.9 (1.81–19.98)
A + I + chemo	3	653	9.47 (6.45–12.49)
Subsequent-line therapy			
A + I	8	198	5.08 (4.01–6.15)
A + I + chemo	2	182	8.13 (5.00–11.26)
Anti-PD-1 therapy			
A + I	7	623	5.90 (5.00–6.79)
A + I + chemo	3	300	8.87 (4.88–12.85)
Anti-PD-L1 therapy			
A + I	4	159	7.07 (3.41–10.72)
A + I + chemo	1	356	8.40 (7.45–9.35)
Antiangiogenic TKIs			
A + I	7	435	5.95 (5.11–6.80)
A + I + chemo	-	-	-
Antiangiogenic mAbs			
A + I	4	94	7.51 (3.14–11.88)
A + I + chemo	4	656	8.78 (6.63–10.93)
EGFR mutation-positive			
A + I	1	40	3.2 (0.75–5.65)
A + I + chemo	2	182	8.13 (5.00–11.26)
EGFR mutation-negative			
A + I	10	742	6.0 (5.34–6.66)
A + I + chemo	3	653	9.47 (6.45–12.49)

95% CI, 95% confidence interval; A + I + chemo, antiangiogenic agents combined with ICIs with chemotherapy; A + I, antiangiogenic agents combined with ICIs; ICIs, immune checkpoint inhibitors; Anti-PD-1, programmed cell death protein-1 inhibitor; Anti-PD-L1, programmed cell death ligand-1 inhibitor; mAbs, monoclonal antibodies; TKIs, tyrosine kinase inhibitors.

Two studies in the A + I + chemo group involving 286 patients reported mPFS according to the PD-L1 expression level in tumors. Compared to the bevacizumab plus chemotherapy arm, mPFS values in the A + I + chemo arm were 10.14 vs. 7.56, 8.58 vs. 7.24, and 10.95 vs. 6.85 months in patients with PD-L1 expression levels of <1%, 1%–49%, and >50% and hazard ratios (HRs) of 0.66 (95% CI, 0.45–0.88), 0.58 (95% CI, 0.43–0.73), and 0.45 (95% CI, 0.30–0.60), respectively. The mPFS for EGFR mutation positive patients treated with A + I + chemo was 8.13 months (95% CI, 5–11.26). The mPFS for EGFR mutation negative patients with A + I + chemo versus A + I was 9.47 months (95% CI, 6.45–12.49) versus 6.0 months (95% CI, 5.34–6.66).

In addition, there were two RCTs in the A + I + chemo group that reported a subgroup analysis with respect to PFS in patients with liver metastases at baseline, which resulted in a pooled HR of 0.43 (95% CI, 0.26–0.60). Ten studies reported the OS data, but the 95% CI upper limit was not reached in four of these studies. Thus, the pooled mOS was also calculated using a weighted average of the single-study medians. The last estimated pooled mOS was 18.6 months. Stratification analysis showed that the mOS values were 21.9 and 14.8 months in the A + I + chemo and A + I subgroups, respectively (Supplementary Table S2).

#### PFS and OS rates

The overall pooled six- and 12-month PFS rates were 64.8% (95% CI, 49.4–80.1) and 45.5% (95% CI, 35.9–55.1), respectively, with 66.9% and 45.8% in the A + I + chemo group and 64.2% and 45.6% in the A + I group, respectively. Five studies documented the 12-month OS rate, and four reported the 18-month OS rate. The overall pooled 12- and 18-month OS rates were 65.4% and 51.0%, respectively, with 74.2% and 54.4% in the A + I + chemo group and 60.9% and 49.7% in the A + I group, respectively. The detailed results are summarized in Supplementary Tables S3, S4.

### Safety

#### Non-hematological AEs

The most common AEs documented in the enrolled studies were proteinuria, hypertension, and rash (Table 4). The pooled frequencies for proteinuria of any grade and of grade ≥ 3 were 38.2% and 4.1%, respectively, with 53.0% and 5.5% in the A + I group and 18.1% and 3.4% in the A + I + chemo group. The pooled frequencies for hypertension of any grade and of grade ≥ 3 were 35.3% and 13.7%, respectively, with 40.0% and 16.2% in the A + I group and 21.1% and 9.7% in the A + I + chemo group. The pooled frequencies for rash of any grade and of grade ≥ 3 were 25.4% and 1.9% overall, 27.9% and 1.2% in the A + I group, and 21.2% and 2.8% in the A + I + chemo group.

**TABLE 4:** Adverse events.

Events	No. of studies	Grade	Incidence, %		
			Overall (%)	A + I group	A + I + chemo group
Proteinuria	5	Any grade	38.2	53.0%	18.1%
	5	Grade ≥ 3	4.1	5.5%	3.4%
Hypertension	9	Any grade	35.3	40.0%	21.1%
	9	Grade ≥ 3	13.7	16.2%	9.7%
Rash	8	Any grade	25.4	27.9%	21.2%
	6	Grade ≥ 3	1.9	1.2%	2.8%
Anaemia	4	Any grade	25.7	25.8%	25.8%
	2	Grade ≥ 3	5.8	0	5.8%
Decreased platelet count	4	Any grade	18.6	20.9%	17.4%
	4	Grade ≥ 3	3.1	1.0%	5.4%
Decreased white blood cell count	5	Any grade	16.5	16.1%	17.0%
	5	Grade ≥ 3	3.3	1.1%	7.4%
Decreased neutrophil count	4	Any grade	17.0	19.1%	12.2%
	4	Grade ≥ 3	3.9	2.1%	8.7%
AST increased	5	Any grade	28.5	34.7%	5.1%
	4	Grade ≥ 3	1.1	1.1%	1.0%
Peripheral neuropathy	2	Any grade	30.1	NR	30.1%
	2	Grade ≥ 3	1.5	NR	1.5%
Decreased appetite	5	Any grade	29.7	34.1%	25.6%
	2	Grade ≥ 3	2.7	0	2.7%
Constipation	2	Any grade	23.6	NR	23.6%
	2	Grade ≥ 3	1.1	NR	1.1%

A + I + chemo, antiangiogenic agents combined with ICIs with chemotherapy; A + I, antiangiogenic agents combined with ICIs; NR, not reported.

Several other toxicities, including peripheral neuropathy, decreased appetite, and constipation, were also reported. Both peripheral neuropathy and constipation were observed only in the A + I + chemo group, while the incidence values for any grade and grade ≥ 3 were 30.1% and 1.5%, respectively, for peripheral neuropathy, and 23.6% and 1.1%, respectively, for constipation. The A + I group had a higher rate for decreased appetite of any grade than the A + I + chemo group (34.1% vs. 25.6%). The incidence of a decreased appetite of grade ≥ 3 was only recorded in the A + I + chemo group, with a value of 2.7%. An increase in aspartate aminotransferase (AST) was also reported. However, the increase in the incidence for AST of any grade was higher by almost 30% among patients in the A + I group than among those in the A + I + chemo group. There was no significant difference in the incidence for AST of grade ≥ 3 observed between the two subgroups.

#### Hematological AEs

Hematological toxicity of grade 3 or higher more commonly occurred in the A + I + chemo group than in the A + I group, including anemia, decreased neutrophil count, decreased white blood cell count, and decreased platelet count. The pooled rates for the above-mentioned hematological AEs of grade ≥ 3 were 5.8% vs. 0, 8.7% vs. 2.1%, 7.4% vs. 1.1%, and 5.4% vs. 1.0% in the A + I + chemo and A + I groups, respectively.

### Sensitivity analysis, publication bias, and meta-regression

**FIGURE 4:**
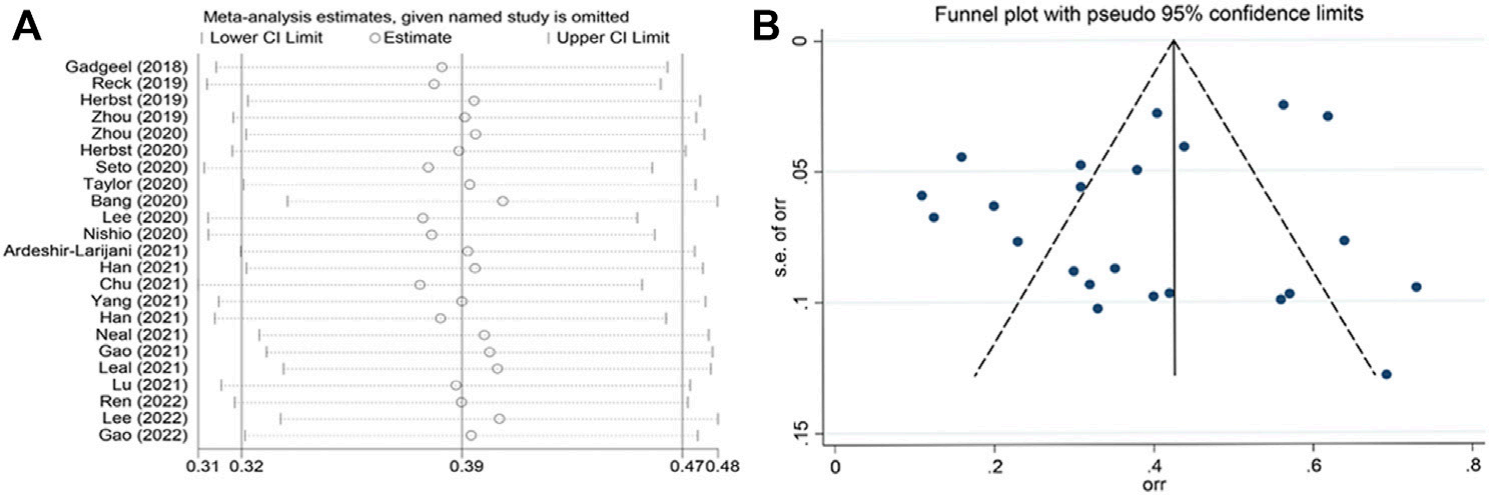
Sensitivity analyses **(A)** and funnel plot **(B)** of the ORR among the included studies.

Sensitivity analyses for the ORR using the leave-one-out approach did not alter the results (Figure 4A). Funnel plots with ORR as the outcome were used to access potential publication bias (Figure 4B). The funnel plots seemed asymmetrical, however, the *p* value of Egger’s test is 0.196, indicating no publication bias among included studies. The adjusted effect yielded by the trim and fill method was the same to the original effect, suggesting no missing studies. In meta-regression, only the proportion of patients with ECOG score 0 in the study population was found to have a significant effect on the pooled adjusted ORR (95% CI, 0.084 to 0.782; *p* = 0.014). Further analyses found no significant effect for age, sample size, sex, smoking history, and tumor histology. The regression data is reported in Supplementary Table S6.

## Discussion

The combination of immunotherapy and antiangiogenic therapy has recently emerged as a novel treatment strategy for the treatment of multiple advanced malignant solid tumors, such as hepatic cell carcinoma (bevacizumab plus atezolizumab), renal cell carcinoma (lenvatinib plus pembrolizumab), and NSCLC [atezolizumab, bevacizumab, carboplatin, and paclitaxel (ABCP)] (Choueiri et al., 2018; Makker et al., 2019; Nishio et al., 2020; Song et al., 2020). Although several large scale prospective RCTs have been conducted evaluating the efficacy and safety of combining immunotherapy, antiangiogenic therapy, and chemotherapy for patients with recurrent or metastatic NSCLC (Socinski et al., 2018; Lee et al., 2020), results from most of these trials are still immature. Our pooled analysis based on 23 prospective studies indicates that combining ICIs with antiangiogenic agents with or without chemotherapy can provide a promising and durable clinical benefit, as well as a favorable safety profile. The data has shown similar mPFS and proportion of patients achieving a response in A + I and A + I + chemo subgroups under first-line treatment setting, with lower frequencies of grade 3–4 AEs observed in the A + I group than in the A + I + chemo group. Moreover, in subsequent-line setting, A + I + chemo treatment showed superior ORR and mPFS over A + I treatment. Although more data from phase III clinical trials are needed to confirm these findings, this meta-analysis attempted to address several controversial issues.

The first question is whether A + I + chemo combination strategy can become the preferred first-line treatment for advanced non-squamous NSCLC. Platinum-based chemotherapy in combination with bevacizumab has been the standard first-line treatment for patients with recurrent or metastatic non-squamous NSCLC (Reck et al., 2016; Mok et al., 2019) until ICI-based therapy became a new first-line treatment option for non-squamous NSCLC without oncogenic driver mutations (Gandhi et al., 2018; Paz-Ares et al., 2018; Socinski et al., 2018). Two phase III RCTs (IMpower150 and TASUKI-52) showed improved PFS with A + I + chemo over A + chemo regardless of PD-L1 expression (Socinski et al., 2018; Lee et al., 2020). Our pooled analysis indicated that the first-line A + I + chemo treatment achieved an ORR, mPFS, and estimated OS of 59%, 9.5 months, and 21.9 months, respectively, in unselected PD-L1 patients. These values are marginally higher to those reported in previous landmark phase III trials that evaluated first-line ICIs as either monotherapy or combination treatment (Supplementary Table S5).

The survival benefits of A + I + chemo combination therapy appeared to be more pronounced in certain population. Previous studies on ICIs have shown minimal therapeutic benefit as a single-agent therapy or in combination with chemotherapy (IMpower130; IMpower132) in patients with baseline liver metastases (West et al., 2019; Nishio et al., 2021). The poor response might be due to tissue-specific immunoregulation and might be reversed by the addition of bevacizumab (Sandler et al., 2006; Facciabene et al., 2011; Tumeh et al., 2017; Pao et al., 2018). The pooled HR for PFS reached 0.43 (95% CI, 0.26–0.60) in patients with liver metastases at baseline from two RCTs (IMpower150; TASUKI-52). In the IMpower150 study, patients with baseline liver metastases had improved OS with ABCP vs. BCP treatment, with an mOS of 13.2 months for ABCP vs. 9.1 months for BCP (HR, 0.67; *p* < 0.01). In the TASUKI-52 study, a trend of improved PFS values was also noted in patients with liver metastases in the nivolumab arm compared to the placebo arm, with an HR of 0.55 (Lee et al., 2020). ICI monotherapy has also demonstrated limited activity in EGFR-mutated NSCLC and the combination of immunotherapy and targeted agents has raised safety concerns. The data from the IMpower150 study suggested an improvement in PFS and OS with the ABCP regimen in EGFR-TKI-resistant NSCLC patients compared to the BCP regimen. Recently, the interim analysis of a phase III ORIENT-31 study demonstrated a significant improvement in mPFS (6.9 vs. 4.3 months) and ORR (44% vs. 25%) with the combination of sintilimab, bevacizumab, pemetrexed and cisplatin compared to pemetrexed plus cisplatin in EGFR-TKI-resistant patients, which further confirms the role of antiangiogenic agents with ICI combined with chemotherapy in EGFR-TKI-resistant patients (Lu et al., 2022). A final OS analysis is eagerly awaited to confirm whether the PFS improvement can translate into a long-term survival benefit. Moreover, a favorable mPFS of 8.1 months (95% CI, 5.00–11.26) was observed in EGFR-mutated patients treated with A + I + chemo therapy from our study.

In summary, based on our meta-analysis, we recommended a combination of ICIs, antiangiogenic agents, and chemotherapy as the preferred first-line treatment for a selected group of patients with limited proven treatment options, such as patients with negative or low PD-L1 expression, liver metastases at baseline, or those with positive EGFR mutations who have failed prior targeted therapy.

The second issue to be addressed was the question of whether the chemo-free strategy of combined ICIs and antiangiogenic agents could be brought into the frontline setting for advanced NSCLC patients, especially for those who cannot tolerate or refuse chemotherapy. Patients treated with first-line A + I therapy alone in our pooled analysis showed an ORR (52% vs. 59%), DCR (85% vs. 89%), and mPFS (10.9 vs. 9.47 months) comparable to those administered first-line A + I + chemo therapy, which were also not inferior to the results of many phase III trials evaluating ICI plus chemotherapy, and even better than historical results for ICI monotherapy (Reck et al., 2016; Paz-Ares et al., 2018; Mok et al., 2019; West et al., 2019; Garassino et al., 2020; Jotte et al., 2020; Nishio et al., 2021). Similarly, a recent real-world study of 69 advanced PD-L1 unselected NSCLC patients showed that first-line A + I therapy resulted in an ORR of 59% (95% CI, 32.7–84.9) and a mPFS of 13.1 months (95% CI, 9.0–17.2) (Qiu et al., 2020). These findings suggest that the chemo-free A + I therapy may provide a new treatment option for advanced NSCLC patients. However, a recently reported phase III LEAP-007 study showed no OS benefit with first-line pembrolizumab plus lenvatinib compared with pembrolizumab alone in patients with PD-L1-positive NSCLC (Yang et al.,2021). Notably, much higher grade 3–5 treatment-related AEs (58% vs. 24%) were reported in the combination group than in the pembrolizumab alone group. Several large-scale prospective RCTs investigating the combination of antiangiogenic agents and immunotherapies in NSCLC are also underway to validate whether or not the chemo-free A + I regimens can be as effective as immunochemotherapy (NCT03976375, NCT04239443, NCT03829332, NCT03516981, NCT02681549).

ICI monotherapy is the current second-line standard treatment if the patients do not receive immunotherapy in the first-line setting. In fact, even as a subsequent-line treatment, A + I therapy seems to confer a certain synergistic effect. Our pooled analysis showed that the A + I in subsequent-line treatment demonstrated an improved one-year OS rate of 58% in patients with unselected histology, which was superior to the pooled results of the CheckMate 017 and 057 studies, with an estimated one-year OS rate of 48% in patients with nivolumab as a subsequent-line treatment (Vokes et al., 2018). Additionally, our analysis showed that subsequent-line A + I treatment resulted in an ORR, PFS, and OS of 22%, 5.1 months and 15.6 months, respectively, which were not inferior to previous RCT studies using ICIs alone in chemotherapy-pretreated and immunotherapy-naïve NSCLC patients (ORRs: 13%–20%, PFSs: 2.3–7.8 months, and OSs: 9.2–13.8 months) (Fehrenbacher et al., 2016; Herbst et al., 2016; Rittmeyer et al., 2017; Vokes et al., 2018). Therefore, A + I also represented a promising treatment strategy for patients who progressed from prior ICI-naïve therapies. In subsequent-line setting, no significant improvement was found in the mPFS of 5.34 months (95% CI, 4.28–6.41 months) in patients without EGFR mutations, and 3.2 months (95% CI, 0.75–5.65 months) in patients with EGFR mutations. However, the inclusion of only one study in the EGFR-mutated subgroup introduced significant statistical bias. We are looking forward to randomized phase III clinical trials enrolling EGFR-mutated patients to validate the results.

It was also important to identify patients who may benefit the most from A + I ± chemo treatment. However, few studies have identified efficacy predictors of A + I therapy (Hegde et al., 2013; Nishino et al., 2017; Mok et al., 2019). In our pooled analysis, improved ORR (47% vs. 28%) was observed for the PD-L1-positive tumors compared to PD-L1-negative tumors in A + I combination trials. Interestingly, in the first-line A + I + chemo group, stratification analysis using PD-L1 expression showed comparable PFS across all categories of tumor PD-L1 expression levels (<1%, 1%–49%, and >50%; median 10.1, 8.6, and 10.9 months), which were better than those in the control arm (median 7.6, 7.2, and 6.9 months). Based on the above analysis, PD-L1 expression cannot be claimed as the efficacy predictor of A + I + chemo.

Given that severe AEs may deteriorate treatment compliance, the tolerability of A + I ± chemo regimen is also worth investigating. Our pooled analysis indicated that a combination of ICIs and antiangiogenic agents has a better safety profile compared to combination therapy with chemotherapy. The grade ≥ 3 AEs especially the hematological toxicity in the A + I group was relatively lower compared to those caused by chemotherapy ± ICIs as previously reported (Supplementary Table S5). Although a higher incidence of AEs of grade ≥ 3 was observed in patients with the combination treatment compared to the ICI monotherapy, most of the AEs were grade 1/2 and well-tolerated. Furthermore, a significantly higher pooled rate of grade ≥ 3 treatment-related adverse effects (TRAEs) with TKIs was observed than with mAbs in the A + I group (62% vs. 34%), which may be attributed to the multitargeting characteristic of TKIs compared to mAbs (Lin et al., 2018). As discussed above in the LEAP-007 study, the median OS was not improved with pembrolizumab plus lenvatinib vs. pembrolizumab, which may have resulted from treatment compliance deterioration due to the high rate of treatment-related AEs (grade 3–5: 57.9%, grade 5: 5.2%), which were mainly hypertension and proteinuria. Similarly, hypertension and proteinuria were also the most common TRAEs observed in another two TKIs (anlotinib and apatinib) (Zhou et al., 2020; Chu et al., 2021). Therefore, A + I regimen should be applied with caution to minimize or reduce the risk of intolerable AEs that might lead to termination of treatment.

Our pooled analysis has several limitations. First, seven phase III RCTs were included, and the majority of the included studies belonged to the single-arm trial and lacked a comparative control group. Second, the results were pooled from heterogeneous studies with different treatment regimens and populations, thus, resulting in unstable merged findings. Therefore, a well-designed randomized control trial with a large sample number is needed to further verify the efficacy of A + I therapy. Finally, due to the limited data and discrepancies in the results with different endpoints, we did not recognize a superiority or inferiority between mAbs and TKIs given as part of combination therapy with immunotherapy based on stratification analysis of the antiangiogenic agent type. A further investigation is thus needed.

## Conclusion

To the best of our knowledge, this is the first pooled analysis evaluating the efficacy and safety of A + I therapy in different treatment lines for patients with NSCLC. The preliminary results showed encouraging antitumor activity and an acceptable toxicity profile for ICIs combined with antiangiogenic agents both as first-line or subsequent-line treatment in patients with advanced NSCLC, making it a promising chemotherapy-free option for both treatment-naïve or pretreated patients, especially those who cannot tolerate chemotherapy. Furthermore, A + I + chemo may also be a promising option for patients with EGFR-TKI resistance or baseline liver metastases. Given that higher incidence of grade ≥ 3 TRAEs was observed with TKIs compared to mAbs in our study, it is worth investigating whether mAbs targeting VEGF or VEGFR are better candidates administered as part of a combination therapy. More in-depth research is needed to explore efficient predictive biomarkers for A + I therapy.

## Data Availability

The original contributions presented in the study are included in the article/Supplementary Material, further inquiries can be directed to the corresponding author.
