# Supplementary material for: Efficacy and safety of combined immunotherapy and antiangiogenesis with or without chemotherapy for advanced non-small-cell lung cancer: A systematic review and pooled analysis from 23 prospective studies
Source: Front Pharmacol. 2022 Aug 10;13:920165. doi: 10.3389/fphar.2022.920165 (PMC9399640; doi:10.3389/fphar.2022.920165)
Supplement: Supplementary file 1 [file DataSheet1.docx]

**Supplementary TABLE 1.** Disease control rate (DCR) for combined immunotherapy and antiangiogenesis therapy with or without chemotherapy.

| **Group** | **No. of studies** | **No. of patients** | **Pooled DCR (95%CI), %** |
| --- | --- | --- | --- |
| **Overall** | 15 | 509 | 83.0 (78.0- 87.0) |
| A+I | 12 | 175 | 81.0 (77.0-86.0) |
| A+I+chemo | 3 | 68 | 89.0 (79.0-98.0) |
| **First-line therapy** |  |  |  |
| A+I | 5 | 169 | 85.0 (78.0-93.0) |
| A+I+chemo | 3 | 68 | 89.0 (79.0-98.0) |
| **Subsequent-line therapy** |  |  |  |
| A+I | 7 | 340 | 78.0 (72.0-85.0) |
| A+I+chemo | - | - | - |
| **Anti-PD-1 therapy** |  |  |  |
| A+I | 8 | 359 | 84.0 (78.0-89.0) |
| A+I+chemo | 2 | 38 | 85.0 (69.0-1.01) |
| **Anti-PD-L1 therapy** |  |  |  |
| A+I | 4 | 150 | 76.0 (65.0-87.0) |
| A+I+chemo | 1 | 30 | 93.0 (84.0-1.02) |
| **Antiangiogenic TKIs** |  |  |  |
| A+I | 8 | 374 | 82.0 (76.0-87.0) |
| A+I+chemo | 1 | 13 | 92.0 (78.0-1.07) |
| **Antiangiogenic mAbs** |  |  |  |
| A+I | 4 | 105 | 80,0 (68.0-92.0) |
| A+I+chemo | 2 | 55 | 86.0 (70.0-1.02) |
| A+I+chemo, antiangiogenic agents combined with ICIs with chemotherapy; A+I, antiangiogenic agents combined with ICIs; ICIs, immune checkpoint inhibitors; Anti-PD-1, programmed cell death protein-1 inhibitor; Anti-PD-L1, programmed cell death ligand-1 inhibitor; mAbs, monoclonal antibodies; TKIs, small molecule tyrosine kinase inhibitors; DCR, disease control rate. | | | |

**Supplementary TABLE 2.** Estimated overall survival (OS) for combined immunotherapy and antiangiogenesis therapy with or without chemotherapy.

| **Group** | **No. of studies** | **No. of patients** | **Estimated OS, months** |
| --- | --- | --- | --- |
| **Overall** | 10 | 1283 | 18.6 |
| A+I | 7 | 586 | 14.8 |
| A+I+chemo | 3 | 697 | 21.9 |
| **First-line therapy** |  |  |  |
| A+I | 1 | 309 | 14.1 |
| A+I+chemo | 3 | 659 | 21.7 |
| **Subsequent-line therapy** |  |  |  |
| A+I | 6 | 277 | 15.6 |
| A+I+chemo | - | - | - |
| **Anti-PD-1 therapy** | 1 | 309 | 14.1 |
| A+I | 6 | 277 | 15.6 |
| A+I+chemo | 3 | 659 | 21.7 |
| **Anti-PD-L1 therapy** | 5 | 534 | 15.0 |
| A+I | 2 | 52 | 12.4 |
| A+I+chemo | - | - | - |
| **Antiangiogenic TKIs** |  |  |  |
| A+I | 4 | 507 | 14.4 |
| A+I+chemo | - | - | - |
| **Antiangiogenic mAbs** |  |  |  |
| A+I | 3 | 79 | 17.1 |
| A+I+chemo | 3 | 697 | 21.9 |
| 95% CI, 95% confidence interval; A+I+chemo, antiangiogenic agents combined with ICIs with chemotherapy; A+I, antiangiogenic agents combined with ICIs; ICIs, immune checkpoint inhibitors; Anti-PD-1, programmed cell death protein-1 inhibitor; Anti-PD-L1, programmed cell death ligand-1 inhibitor; mAbs, monoclonal antibodies; TKIs, tyrosine kinase inhibitors. | | | |

**Supplementary TABLE 3.** Six-month progression-free survival (PFS) and 12-month PFS rate for combined immunotherapy and antiangiogenesis with or without chemotherapy.

| **Group** | **No. of studies** | **No. of patients** | **Pooled PFS rate (95%CI), %** |
| --- | --- | --- | --- |
| **6-mo PFS rate (overall）** | 5 | 472 | 64.8 (49.4-80.1) |
| A+I+chemo (first-line) | 1 | 356 | 66.9 (62.0-71.9) |
| A+I | 4 | 116 | 64.2 (39.2-89.1) |
| First-line | 2 | 61 | 80.1 (53.6-1.07) |
| Subsequent-line | 2 | 55 | 47.8 (14.5-81.1) |
| PD-1 | 2 | 49 | 79.6 (51.2-1.08) |
| PD-L1 | 2 | 67 | 49.2 (13.9-84.5) |
| TKIs | 1 | 22 | 94.0 (76.1-1.12) |
| mAbs | 3 | 94 | 54.4 (31.3-77.4) |
| PD-1 | 1 | 27 | 65.0 (46.5-83.4) |
| PD-L1 | 2 | 67 | 49.2 (13.9-84.5) |
| **12-mo PFS rate (overall)** | 8 | 817 | 45.5 (35.9-55.1) |
| A+I+chemo (first-line) | 3 | 661 | 45.8 (34.2-57.4) |
| A+I | 5 | 142 | 45.6 (26.2-64.9) |
| First-line | 3 | 88 | 56.9 (46.7-67.1) |
| Subsequent line | 2 | 55 | 25.3 (14.4-36.3) |
| PD-1 | 3 | 75 | 53.1 (35.2-71.1) |
| First-line | 2 | 87 | 56.9 (46.7-67.1) |
| Subsequent-line | 1 | 27 | 43.0 (24.3-61.7) |
| mAbs | 2 | 53 | 44.0 (30.6-57.3) |
| TKIs | 1 | 22 | 71.4 (52.5-90.3) |
| PD-L1 | 2 | 67 | 35.3 (-2.9-73.4) |
| TKIs | 1 | 22 | 71.4 (52.5-90.3) |
| mAbs | 4 | 120 | 39.3 (20.6-58.0) |
| First line | 2 | 65 | 50.9 (38.8-63.0) |
| Subsequent-line | 2 | 55 | 28.7 (2.3-55.1) |
| PD-1 | 2 | 53 | 44.0 (30.6-57.3) |
| PD-L1 | 2 | 67 | 35.3 (-2.9-73.4) |

95% CI, 95% confidence interval; PFS, progression-free survival; A+I+chemo, antiangiogenic agents combined with ICIs with chemotherapy; A+I, antiangiogenic agents combined with ICIs; mAbs, monoclonal antibodies; TKIs, small molecule tyrosine kinase inhibitors.

**Supplementary TABLE 4.** Twelve- and 18-month overall survival (OS) rate for combined immunotherapy and antiangiogenesis with or without chemotherapy.

| **Group** | **No. of studies** | **No. of patients** | **Pooled OS rate (95%CI), %** |
| --- | --- | --- | --- |
| **12-mo OS rate (overall)** | 7 | 645 | 65.4 (57.9-72.8) |
| A+I+chemo (first-line) | 2 | 430 | 74.2 (60.3-88.1) |
| A+I | 4 | 186 | 60.9 (51.2-70.7) |
| First-line | 1 | 26 | 73.0 (55.9-90.1) |
| Subsequent-line | 3 | 160 | 57.6 (48.8-66.4) |
| TKIs | 1 | 164 | 57.0 (47.5-66.5) |
| mAbs | 3 | 81 | 63.3 (48.6-78.0) |
| PD-1 | 3 | 158 | 63.7 (53.6-73.9) |
| PD-L1 | 1 | 28 | 48.0 (29.5-66.5) |
| **18-mo OS rate (overall)** | 4 | 481 | 51.0 (37.5-64.6) |
| A+I+chemo (first-line) | 1 | 400 | 54.4 (49.5-59.3) |
| A+I | 3 | 81 | 49.7 (26.1-73.2) |
| First-line | 1 | 26 | 64.0 (45.5-82.5) |
| Subsequent-line | 2 | 55 | 42.7 (11.3-74.1) |
| PD-1 | 2 | 53 | 61.5 (48.4-74.6) |
| PD-L1 | 2 | 28 | 27.0 (10.6-43.4) |

95% CI, 95% confidence interval; OS, overall survival; A+I+chemo, antiangiogenic agents combined with ICIs with chemotherapy; A+I, antiangiogenic agents combined with ICIs; mAbs, monoclonal antibodies; TKIs, small molecule tyrosine kinase inhibitors.

**Supplementary TABLE 5.** Results for landmark phase III trials evaluating immune checkpoint inhibitors in advanced or metastatic NSCLC.

| **Study** | **Treatment line** | **Patients** | **No. of patients** | **Treatment regimen** | **ORR (%)** | **mPFS**  **(months)** | **mOS**  **(months)** |
| --- | --- | --- | --- | --- | --- | --- | --- |
|  |  |  |  |  |  |  |  |
| Checkmate 017 | 2 | Squamous NSCLC | 135  137 | Nivolumab  Docetaxel | 20.0  8.8 | 3.5  2.8 | 9.2  6.0 |
| Checkmate 057 | 2 or 3 | Non-squamous NSCLC | 292  290 | Nivolumab  Docetaxel | 19.2  12.4 | 2.3  4.2 | 12.2  9.4 |
| KEYNOTE-010 | ≥2 | NSCLC with PD-L1 positive | 345  346  343 | Pembrolizumab (2mg/kg) Pembrolizumab (10mg/kg) Docetaxel | 18.0  18.5  9.3 | 3.9  4.0  4.0 | 10.4  12.7  8.5 |
| KEYNOTE-024 | 1 | NSCLC with PD-L1 ≥50% | 154  151 | Pembrolizumab  Chemotherapy | 44.8  27.8 | 10.3  6.0 | 30.0  14.2 |
| KEYNOTE-042 | 1 | PD-L1 positive NSCLC | 299  300  413  405  637  637 | Pembrolizumab^a^  SOC^a^  Pembrolizumab^b^  SOC^b^  Pembrolizumab^c^  SOC^c^ | 39.5  32.0  33.4  28.9  27.3  26.5 | 7.1  6.4  6.2  6.6  5.4  6.5 | 20.0  12.2  17.7  13.0  16.7  12.1 |
| OAK | 2 or 3 | NSCLC | 612  613 | Atezolizumab  Docetaxel | 13.6  13.4 | 2.8  4.0 | 13.8  9.6 |
| POPLAR | 2 or 3 | NSCLC | 144  143 | Atezolizumab  Docetaxel | 17  15 | 7.8  3.9 | 12.6  9.7 |
| KEYNOTE-407 | 1 | Squamous NSCLC | 278  281 | Pembrolizumab +PC  PC | 57.9  38.4 | 6.4  4.8 | 15.9  11.3 |
| KEYNOTE-189 | 1 | Non-squamous NSCLC | 410  206 | Pembro+AC/AP Placebo+AC/AP | 47.6  18.9 | 8.8  4.9 | NA  11.3 |
| IMpower150 | 1 | NSCLC | 402  400  400 | ACP  ABCP  BCP | 40.6  56.4  40.2 | 6.7  8.4  6.8 | 19.4  19.8  14.9 |
| IMpower130 | 1 | Non-squamous NSCLC | 483  240 | Atezo+NabPacC  Nab-PacC | 49.2  31.9 | 7.0  5.5 | 18.6  13.9 |
| IMpower131 | 1 | Squamous NSCLC | 338  343  340 | Atezo+PacC Atezo+NabPacC  Nab-PacC | -  32  16 | NR  6.3  5.6 | NR  14.2  13.5 |
| IMpower132 | 1 | Non-squamous NSCLC | 292  286 | Atezo+Cis/Car +Pem  Cis/Car+Pem | 47  32 | 7.6  5.2 | 18.1  13.6 |
| ^a^Patients with PD-L1 tumor proportion score (TPS) ≥50%; ^b^Patients with TPS ≥20%; ^c^Patients with TPS ≥1%; SOC, standard of care; NC, not calculable; ORR, objective response rate; mPFS, median progression-free survival; ACP, atezolizumab + carboplatin + paclitaxel; ABCP, atezolizumab + bevacizumab + carboplatin + paclitaxel; BCP, bevacizumab + carboplatin + paclitaxel; PC, nab-paclitaxel or paclitaxel + carboplatin; Cis/Car, cisplatin or carboplatin; NR, not reached; NA, not available. | | | | | | | |

**Supplementary TABLE 6.** Univariable Meta-Regression of Covariates against ORR of combined immunotherapy and antiangiogenesis with or without chemotherapy.

| **Variable** | **No. of Studies** | **Regression Coefficient** | **95%CI** | **p** |
| --- | --- | --- | --- | --- |
| Age, median | 22 | .0012516 | -.0054345 to .0079377 | 0.700 |
| Sample size | 23 | .0004714 | -.0002256 to .0011685 | 0.174 |
| Sex (male or female) | 22 | .1198496 | -.3387439 to .5784431 | 0.592 |
| ECOG score (0 or 1) | 22 | .4330404 | .0840296 to .7820512 | 0.018* |
| Smoking history (yes or no) | 19 | -.0013589 | -.4019849 to .3992671 | 0.994 |
| Tumor histology | 23 | -.0511488 | -.2096445 to .1073468 | 0.509 |

CI, confidence interval; *P < 0.05.

**Supplementary TABLE 7.** Baseline characteristics of patients in included studies.

| **Study** | **Year** | **Tumor histology** | **Tx arm**  **(n)** | **Median age, years** | **Stage** | **ECOG performance status, n** | **Smoking status, n** | **EGFR mutation status, n** | **PD-L1 TPS, n** |
| --- | --- | --- | --- | --- | --- | --- | --- | --- | --- |
|  |  |  |  |  | **(Male/Female)** | **0/1** | **Never/Current or previous smoker** | **Negative/Positive/Unknown** | **Positive(≥50%/1%–49%)/Negative** |
| Reck | 2019 | Non-squamous | Atezo+PacCb (n=402) Atezo+Bev+PacCb (n=400) Bev+PacCb (n=400) | 63/63/63 | IV  (241/161  230/160  239/161) | 180/222  159 /238  179/218 | 77/325  82/318  77/323 | 348/45/9  353/34/13  345/45/10 | 68/148/185  75/135/190  73/127/200 |
| Zhou | 2020 | Non-squamous | Cam+Apa (n=105) | 58 | IIIB/IV  (79/26) | 13/92 | 43/62 | 105/0/0 | 3/NR/66 |
| Herbst | 2019 | NSCLC | Pembro +Ram (n=27) | 65 | IV  (21/6) | 7/20 | 1/26 | 23/1/3 | 4/7/11 |
| Herbst | 2020 | NSCLC | Pembro+Ram (n=26) | 63 | III/IV  (12/14) | 11/15 | 2/24 | 26/0/0 | 16/9/0 |
| Chu | 2021 | NSCLC | Sinti+Anlo (n=22) | 64.5 | IIIb/IIIc/IV  (21/1) | 1/21 | 8/14 | 22/0/0 | 8/5/8 |
| Seto | 2020 | NSCLC | Atezo+Bev (n=39) | 67 | III/IV  (33/6) | 25/14 | 3/36 | 39/0/0 | 39/0/0 |
| Lee | 2020 | Non-squamous | Niv+Bev+PacCb (n=275) Placebo+Bev+PacCb (n=275) | 66/66 | IIIb/IV  (205/70  206/69) | 129/128  146/147 | 61/214  54/221 | 275/0/0  275/0/0 | 73/81/120  74/81/120 |
| Taylor | 2020 | NSCLC | Pembro+Lenva (n=21) | 65 | III/IV  (10/11) | 6/15 | NR | NR | 14/5/2 |
| Nishio | 2020 | Non-squamous | Pembro+Lenva+PemCb/Cis (n=13) | 65 | IV  (7/6) | 7/6 | 4 /9 | 13/0/0 | 3/8/2 |
| Bang | 2020 | NSCLC | Durva+Ram (n=28) | 64.5 | IIIb/IV  (19/9) | 16/12 | 6/22 | 23/2/3 | ≥25% 5/<25% 17 (61)/NR |
| Ardeshir-Larijani | 2021 | Non-squamous | Atezo+Bev+PemCb (n=30) | 64 | III  (10/20) | 10/20 | NR | NR/23/NR | 7/14/9 |
| Yang | 2021 | Non-squamous | Pembro+Lenva (n=309) Pembro+Placebo (n=314) | 66/66 | IV  (230/79  224/90) | 110/199  108/206 | 49/255  67/247 | NR | 137/172/0  139/175/0 |
| Ren | 2022 | Non-squamous | Cam+Apa (n=25) | 61 | IIIb/IIIc/IV  (19/6) | 5/20 | 6/19 | 25/0/0 | 4/NR/10 |
| Han | 2021 | Non-squamous | Penpulimab+Anlo (n=26) | 59 | IIIb/IIIc/IV  (20/6) | 20/6 | NR | 26/0/0 | NR |
| Zhou | 2019 | Non-squamous | Cam+Apa (n=96) | 57 | IIIb/IV  (76/20) | 14/82 | 31/54 | 96/0/0 | NR |
| Neal | 2021 | NSCLC | Atezo+cabozantinib (n=30) | 67 | IV  (13/17) | 13/17 | 4/26 | 30/0/0 | 15/3/12 |
| Leal | 2021 | Non-squamous | Nivo+sitravatinib (n=68) | 66 | III-IV  (29/39) | 18/45 | 12/56 | 68/0/0 | NR |
| Han | 2021 | NSCLC | TQ-B2450 (PD-L1)+Anlo (n=68)  TQ-B2450 (PD-L1) (n=33) | 61.5/60 | IIIb/IV  (47/21  27/6) | 3/65  2 /31 | 24/41  11/22 | 68/0/0  33/0/0 | NR |
| Lee | 2022 | NSCLC | Atezo +Bev (n=24) | 63 | IIIb/IV  (13/11) | 0/24 | 13/11 | 24/0/0 | 6/18 |
| Gao | 2021 | NSCLC | Cam+Apa (n=40) | NR | NR | NR | NR | 40/0/0 | NR |
| Gao | 2022 | Non-central squamous | Cam+Apa (n=25) | 63 | IIIb/IV  (23/2) | 1/24 | 2/23 | NR | 11/13 |
| Gadgeel | 2018 | Non-squamous | Pembro+Bev+PacCb (n=25) | 62 | IIIb/IV  (13/12) | 11/14 | 1/24 | 25/0/0 | 8/12/5 |
| Lu | 2021 | Non-squamous | Sinti+Bev+PemCs (n=148)  Sinti+ PemCs (n=145)  PemCs (n=151) | 59/57/56 | IIIb/IIIc/IV | 36/112  22/123  26/125 | 103/45  102/43  107/44 | 0/148/0  0/145/0  0/151/0 | NR |

Tx, treatment; NSCLC, non-small cell lung cancer; Atezo, atezolizumab; Bev, bevacizumab; PacCb, paclitaxel plus carboplatin; Cam, camrelizumab; Apa, apatinib; Pembro, pembrolizumab; Ram, ramucirumab; Sin, sintilimab; Anlo, anlotinib; Niv, nivolumab; Len, lenvatinib; PemCb, pemetrexed plus carboplatin; Durva, durvalumab; Cis, cisplatin; mos, months;
